# Metabolic pathways of *Pseudomonas aeruginosa* involved in competition with respiratory bacterial pathogens

**DOI:** 10.3389/fmicb.2015.00321

**Published:** 2015-04-23

**Authors:** Marie Beaume, Thilo Köhler, Thierry Fontana, Mikael Tognon, Adriana Renzoni, Christian van Delden

**Affiliations:** ^1^Service of Infectious Diseases, Department of Medical Specialities, University Hospitals GenevaGeneva, Switzerland; ^2^Department of Microbiology and Molecular Medicine, University of GenevaGeneva, Switzerland

**Keywords:** direct competition, exploitative competition, respiratory pathogens, *Pseudomonas aeruginosa*, beneficial interactions, *Staphylococcus aureus*

## Abstract

**Background:** Chronic airway infection by *Pseudomonas aeruginosa* considerably contributes to lung tissue destruction and impairment of pulmonary function in cystic-fibrosis (CF) patients. Complex interplays between *P. aeruginosa* and other co-colonizing pathogens including *Staphylococcus aureus*, *Burkholderia* sp., and *Klebsiella pneumoniae* may be crucial for pathogenesis and disease progression.

**Methods:** We generated a library of PA14 transposon insertion mutants to identify *P. aeruginosa* genes required for exploitative and direct competitions with *S. aureus*,* Burkholderia cenocepacia,* and* K. pneumoniae*.

**Results:** Whereas wild-type PA14 inhibited *S. aureus* growth, two transposon insertions located in *pqsC and carB*, resulted in reduced growth inhibition. PqsC is involved in the synthesis of 4-hydroxy-2-alkylquinolines (HAQs), a family of molecules having antibacterial properties, while *carB* is a key gene in pyrimidine biosynthesis. The *carB* mutant was also unable to grow in the presence of *B. cepacia* and *K. pneumoniae* but not* Escherichia coli* and *S. epidermidis*. We further identified a transposon insertion in *purF*, encoding a key enzyme of purine metabolism. This mutant displayed a severe growth deficiency in the presence of Gram-negative but not of Gram-positive bacteria. We identified a beneficial interaction in a *bioA* transposon mutant, unable to grow on rich medium. This growth defect could be restored either by addition of biotin or by co-culturing the mutant in the presence of *K. pneumoniae* or *E. coli*.

**Conclusion:** Complex interactions take place between the various bacterial species colonizing CF-lungs. This work identified both detrimental and beneficial interactions occurring between *P. aeruginosa* and three other respiratory pathogens involving several major metabolic pathways. Manipulating these pathways could be used to interfere with bacterial interactions and influence the colonization by respiratory pathogens.

## Introduction

The composition of the microbial respiratory flora of cystic fibrosis (CF) patients changes with age; *Haemophilus influenzae* and *Staphylococcus aureus* being present mainly in young children, while *Pseudomonas aeruginosa, S. aureus,* and *Burkholderia* sp. predominate in older patients (Goss and Burns, 2007; Cox et al., 2010; Lipuma, 2010). At the age of 20, 60–70% of CF-patients present intermittent colonization by *P. aeruginosa* (Folkesson et al., 2012) which progressively evolves toward a chronic colonization, that entails progressive lung tissue damage and impairment of pulmonary function. The disappearance of the early colonizing species may be explained by antibiotic treatments or by competition between co-colonizing microorganisms. Many ecological factors, including host immune responses as well as the presence of co-colonizing species interacting with* P. aeruginosa* by competition and/or cooperation, determine the dynamics of lung colonization. Bacterial interference occurs either via direct competition characterized by an active mechanism including the type VI secretion systems (Pukatzki et al., 2006; Basler et al., 2013) and production of competitor molecules (siderophores, secondary metabolites, toxins), or via exploitative competition where one organism consumes the resources of another (Cornforth and Foster, 2013; Boon et al., 2014). For example, *P. aeruginosa* can lyse* S. aureus* to obtain iron under iron-starvation conditions using LasA protease (Mashburn et al., 2005), and 4-hydroxy-2-heptylquinoline *N*-oxide (HQNO) allows *P. aeruginosa* to inhibit the cytochrome oxidase of *S. aureus* (Lightbown and Jackson, 1954; Machan et al., 1992; Toder et al., 1994). Furthermore, recent results demonstrated that compounds such as phenazines inhibit methicillin-resistant *S. aureus* (MRSA; Cardozo et al., 2013). Besides active competition, regulatory effects induced for instance by the resident flora can induce the expression of important virulence and metabolic genes in *P. aeruginosa* (Duan et al., 2003). In addition, positive interactions can also occur between microorganisms during polymicrobial infections. For example, *P. aeruginosa* can induce the formation of small colony variants in *S. aureus,* leading to an increased resistance of the latter to aminoglycoside antibiotics (Hoffman et al., 2006). This indicates that interspecies competition and cooperation play an important role in shaping composition and structure of polymicrobial bacterial populations, thereby potentially influencing disease progression. Increasing our understanding of these interactions is crucial (Bragonzi et al., 2012) and might lead to the identification of new targets aiming at manipulating interactions inside polymicrobial communities to the disadvantage of pathogens such as *P. aeruginosa*.

In this perspective we developed a broad genetic screen to identify* P. aeruginosa* genes required for direct and exploitative competition. We searched for *P. aeruginosa* mutants affected in growth in the presence of *S. aureus*, *Klebsiella pneumoniae,* or *B. cepacia*. In addition, we examined *P. aeruginosa* mutants altered in their capacity to inhibit growth of *S. aureus*.

## Materials and Methods

### Bacterial Strains, Growth Conditions and Media

All *P. aeruginosa* mutants used in this study are derived from the clinical strain PA14 (Rahme et al., 1995; **Table 1**). All genetic manipulations were performed in *Escherichia coli* strain DH10B. *E. coli* strain SM10-check λpir was used for conjugating plasmids into *P. aeruginosa.* Three bacterial pathogens were used for the initial screening: the *S. aureus* clinical strain COL (Dyke et al., 1966), a clinical *Burkholderia cenocepacia* isolate (University Hospitals of Geneva) and a non-capsulated *K. pneumoniae* strain (laboratory collection). The *S. epidermidis* and the *E. coli* strains were isolated from tracheal aspirates of two CF patients followed at the University Hospitals of Geneva (Geneva, Switzerland).

**Table 1:** Plasmids and strains.

Plasmids or strains	Description	Reference
**Plasmids**
pIApX2	Broad-host-range cloning vector, pUCP20 derivative	I. Attree (Grenoble, France; West et al., 1994)
pME3087	Mobilizable suicide vector, ColE1	Voisard et al. (2007)
pMMB207	Expression plasmid with inducible tac/lacUV5 promoter	Morales et al. (1991)
pPqsCDE	*pqsCDE* PCR fragment cloned into pIApX2 by using XbaI (Roche) and HindIII (Roche)	This study
pCarB	*carB* PCR fragment cloned into pIApX2 by using HindIII (Roche) and BamHI (Roche)	This study
pBioA	*bioA* PCR fragment cloned into pMMB207 by using KpnI (NEB) and BamHI (Roche)	This study
pPurF	*purF* PCR fragment cloned into pMMB207 by using KpnI (NEB) and BamHI (Roche)	This study
**Strains**
PA14	Clinical isolate UCBPP-PA14	Rahme et al. (1995)
PA14 *carB*::Tn	Transposon inserted into the *carB* gene	This study
PA14 *pqsC*::Tn	Transposon inserted into the *pqsC* gene	This study
PA14 *purF*::Tn	Transposon inserted into the *purF* gene	This study
PA14 *bioA::*Tn	Transposon inserted into the *bioA* gene	This study
PA14 *carB*::Tn pCarB	*carB*-disrupted* Pseudomonas aeruginosa* containing the pCarB complementation plasmid	This study
PA14 *pqsC*::Tn pPqsCDE	*pqsC*-disrupted* P. aeruginosa* containing the pPqsCDE complementation plasmid	This study
PA14 *purF*::Tn pPurF	*purF-*disrupted* P. aeruginosa* containing the pPurF complementation plasmid	This study
PA14 *bioA*::Tn pBioA	*bioA-*disrupted* P. aeruginosa* containing the pBioA complementation plasmid	This study
PA14 *carB*::Tn pIApX2	*carB*-disrupted* P. aeruginosa* containing the pIApX2 plasmid	This study
PA14 *pqsC*::Tn pIApX2	*pqsC*-disrupted* P. aeruginosa* containing the pIApX2 plasmid	This study
PA14 *purF::*Tn pMMB207	*purF-*disrupted* P. aeruginosa* containing the pMMB207 plasmid	This study
PA14 *bioA::*Tn pMMB207	*bioA-*disrupted* P. aeruginosa* containing the pMMB207 plasmid	This study
PA14 Δ *lasA*	*LasA*-deleted* P. aeruginosa* PA14 strain	This study
PAO1 Δ *pqsA pqsA*::*lux*	*PqsA*-deleted *P. aeruginosa* PAO1 strain with a copy of the *pqsA* promoter linked to the *luxCDABE* genes and inserted into a neutral site in the chromosome	Fletcher et al. (2007)
PA14* mvfR^-^*	Mutated PA14 strain containing a nonsense point mutation in *mvfR*	Cao et al. (2001)
PA14* pqsA^-^*	Mutated PA14 strain containing a non-polar deletion of *pqsA*	Deziel et al. (2004)
PA14 *pqsH*^-^	Mutated PA14 strain containing a *aacC1* cassette inserted into *pqsH*	Xiao et al. (2006a)
PA14 *pqsL*^-^	Isogenic non-polar* pqsL* deletion mutant	Lepine et al. (2004)
*Escherichia coli* SM10-check λ*pir*	RP4-2-Tc::Mu *recA, pir* lysogen KmR	Simon et al. (1983)
*Staphylococcus aureus* COL	Clinical strain, MRSA	Dyke et al. (1966)
*Burkholderia cenocepacia*	Clinical strain	University Hospitals of Geneva
*Klebsiella pneumoniae*	Non-capsulated	Laboratory collection
*E. coli*	Clinical strain from CF-patient	This study
*S. epidermidis*	Clinical strain from CF-patient	This study

When required, plates were supplemented with 1 mM uracil, 1 mM arginine, or 2 mg/L biotin (final concentrations). For growth under hypoxic conditions, LB plates were supplemented with 5 mM KNO_3_ and incubated in a jar using the GasPak Plus kit (BBL) (final O_2_ concentration < 0.2%).

### Generation of the *P. aeruginosa* PA14 Transposon (Tn) Mutant Library

The *E. coli* SM10-check λ*pir* donor strain containing pBT20 (Kulasekara et al., 2005) and the recipient *P. aeruginosa* PA14 were grown overnight at 37°C with shaking (250 rpm) in LB-broth. 200 μL of the donor and 100 μL of the recipient culture were mixed and centrifuged at 6,000 rpm for 2 min. The pellet was resuspended in 20 μL of LB and deposited on an LB agar plate. After 5 h of incubation at 37°C, cells were resuspended in 1 mL 0.9% NaCl and 100 μL of the suspension were plated on LB agar medium containing gentamicin (50 mg/L) and chloramphenicol (10 mg/L). To test for random distribution of Tn-insertions, 200 transconjugants were picked and streaked on minimal M9-salts medium supplemented with 0.2% glucose and 1 mM MgSO_4_ and on an LB-agar plate. Approximately 1% of clones were auxotrophic, which is agreement with a random transposition event.

### Growth Competition Screening

Overnight cultures of bacterial competitors were adjusted to OD_600_ = 0.5. A 500 μL aliquot of fivefold (*S. aureus* and *S. epidermidis*) and 100-fold (*B. cepacia*, *K. pneumoniae,* and *E. coli*) dilutions were plated on agar plates. Bacterial lawns were incubated 2 h at 37°C. Overnight cultures of *P. aeruginosa* transposon mutants were adjusted to OD_600_ = 2 and 1 μL of each culture was spotted on the competitor lawns using a 48-pin inoculator. As control, cells were spotted on a plate without competitors. Plates were incubated 18 h at 37°C. Mutants were initially screened for: (i) an alteration of the inhibition zone on the *S. aureus* lawn on Mueller Hinton agar, and (ii) an alteration of *P. aeruginosa* growth on lawns of *B. cepacia* and *K. pneumoniae* on LB-agar.

### Identification of the Transposon Insertion Site

The transposon insertion site was identified by a two-step semi-random PCR (Friedman and Kolter, 2004; Kulasekara et al., 2005). The first round PCR reaction contained dNTPs (0.25 mM final), Taq Buffer (1X final, Sigma), DMSO (5% final concentration), Taq polymerase (2.5 U/reaction, Sigma), and primers MCL195 and ARB1 (**Table 2**). Four microliters of bacterial lysate served as template. PCR cycling conditions were as follows: 95°C for 3 min, 36 cycles of 95°C for 20 s, 30 to 48°C for 40 s with a 0.5°C increase per cycle, 72°C for 1 min and a final extension at 72°C for 5 min. In the second round PCR reaction the nested primers ARB2 and MCL210 were used (**Table 2**). PCR conditions were as follows: 95°C for 3 min, 30 cycles of 95°C for 30 s, 50°C for 30 s, 72°C for 1 min and a final extension of 72°C for 4 min. PCR products were visualized on a 1.6% agarose gel. The most intense bands were extracted and purified (GeneJet Gel extraction kit, ThermoScientific). The PCR products were then submitted to DNA sequencing (Fasteris SA, Geneva, Switzerland) using the nested primers ARB2 and MCL210. Each sequence was aligned to the PA14 genome sequence in the *Pseudomonas* database (Winsor et al., 2011). Confirmation of the transposon insertion was done by PCR using gene and/or transposon specific primers. PCR cycling conditions were: (i) activation: 95°C for 2 min, (ii) denaturation: 95°C for 20 s, annealing: 57°C for 30 s, extension: 72°C for 1 min and 30 s, (iii) final extension: 72°C for 4 min. Step 2 was repeated 27 times. PCR products were purified and submitted to DNA sequencing.

**Table 2:** Primers used in this study

Primers	Amplified gene(s)	Sequences (5^′^–3^′^)	Final concentration (μM)
**Semirandom PCR**
ARB1		GGCCACGCGTCGACTAGTACNNNNNNNNNNGATAT	0.5
ARB2		GGCCACGCGTCGACTAGTAC	0.4
MCL195		GATCCCGCAGTGGCTCTCTATACAAAGTTG	0.2
MCL210		TGGTGCTGACCCCGGATGAAG	0.4
**Confirmation of the transposon insertion site and complementations^∗^**
PurF-Kpn	*purF*	ACAGGTACCAGTGATTTTGGCGGGACAC	0.6
PurF-BamHI		ACAGGATCCCCAGGGTGTCGAAGGCC	0.6
BioA-Kpn	*bioA*	ACAGGTACCTGAACACCCCCAACATGAGA	0.6
BioA-R_BamHI		ACAGGATCCGATTCGAGGGTAGTGGCGAC	0.6
CarB-F_BamHI	*carB*	ACAGGATCCGTTCGTCGATCCCGGCTA	0.6
CarB-R_HindIII		ACAAAGCTTTCGGCGTTTTCCTTGAGG	0.6
pqsC-Xba	*pqsCDE*	ACACTCTAGATTCGAACTGGCGTCGCAAC	0.6
pqsC-Hind		ACACAAGCTTTCTTCCAGTCGATAGCCAACC	0.6
***lasA* deletion^∗^**
LasAF-Eco	5^′^ *lasA* region	CCCGGAATTCAGGATAACGTCGGCATGGAC	0.6
LasAR-Bam		CCGCGGATCCTGCTCCAGGTATTCGCTCTTG	0.6
LasAF-Bam	3^′^ *lasA* region	CCGCGGATCCACCAGATCCAGGTGAGCAACG	0.6
LasAR-Hind		CCCCAAGCTTTCGGAGTCCGGCTACTACGC	0.6

^∗^Restriction enzyme recognition sites are underlined

### Exoproduct Analysis

Rhamnolipid production was assessed following a previously described protocol (Kohler et al., 2000). Plates were incubated 18 h at 37°C, 24 h at room temperature and then 16 h at 4°C. The diameter of the rhamnolipid-containing halo formed around the bacterial colony was measured and compared with that produced by the reference strain PA14. Determinations were done in duplicates.

Elastase activity was determined using the Elastin Congo Red assay with modifications (Pearson et al., 1997). Overnight cultures of *P. aeruginosa* strains were prepared in LB medium supplemented with 1 mM uracil to optimize the growth of the strain PA14 *carB*::Tn. Cultures were grown for 7 h at 37°C in PB medium (Essar et al., 1990). The OD_600_ was measured and elastase activity determined in supernatants using the Elastin Congo Red assay measuring absorption at OD_495_. All determinations were done in triplicate and expressed as the ratio of OD_495_/OD_600_.

### Extra-Chromosomal Complementations

The coding regions of *carB*, *pqsCDE*,* bioA,* and* purF* including 100 nucleotides upstream of the start codon were amplified by PCR from PA14 genomic DNA using the primers listed in **Table 2** and following the same PCR conditions as described above. PCR products were purified and cloned into vectors pIApX2 (*pqsCDE*, *carB*) or pMMB207 (*purF*, *bioA*; Morales et al., 1991; West et al., 1994; **Table 1**). 1.5 μg of each vector and insert were digested with the appropriate restriction enzymes and buffers in a final volume of 25 μL (**Table 1**). After purification on agarose gel (GeneJet Gel extraction kit, ThermoScientific), vectors and inserts were ligated (T4 DNA ligase, Promega). *E. coli* DH10B thermo-competent cells were transformed with the ligation mixture and transformants selected on LB plates supplemented with 100 μg/mL ampicillin (*pqsCDE*, *carB*) or 15 μg/mL chloramphenicol (*purF*, *bioA*). The resulting plasmids pCarB and pPqsCDE were electroporated into the corresponding PA14 Tn-mutants and selected on LB-agar plates supplemented with 200 μg/mL carbenicillin. Plasmids pPurF and pBioA were transferred into the corresponding PA14 Tn-mutants by triparental mating using pRK2013 as a helper plasmid (Figurski and Helinski, 1979). Transconjugants were selected on LB-agar plates supplemented with 250 μg/mL chloramphenicol and 25 μg/mL gentamycin to counterselect *E. coli* donor strains.

### Construction of the *P. aeruginosa lasA* Mutant

A *lasA* deletion mutant was constructed by homologous recombination using plasmid pME3087 (Voisard et al., 2007). For the 5^′^-region PCR fragment, we used dNTP (0.2 mM final), Taq Buffer (1X final, Sigma), DMSO (5% final), Taq polymerase (2.5 U/reaction), and 4 μL of PA14 lysate. PCR conditions were as follows: one cycle of 2 min at 95°C, 27 cycles of 20 s at 95°C, 30 s at 57°C, 1 min at 72°C followed by a final extension of 4 min at 72°C. For the 3^′^-region PCR fragment, we used dNTP (0.2 mM final), PFU Buffer (1X final), DMSO (5% final), PFU polymerase (2.5 U/reaction), and 4 μL of PA14 bacterial lysate. PCR conditions were as follows: one cycle of 95°C for 2 min, 30 cycles of 20 s at 95°C, 30 s at 55°C, 1 min at 72°C, followed by a final extension of 4 min at 72°C. The 5^′^-region and the 3^′^ region fragments were digested with BamHI. Both fragments were ligated and PCR re-amplified using primers LasAF-Eco and LasAR-Hind (**Table 2**). The generated 1.6 kbp fragment was digested using *Eco*RI and *Hin*dIII restriction enzymes and cloned into plasmid pME3087. The resulting plasmid plasA1 was introduced into *E. coli* strain SM10lpir for subsequent conjugation into *P. aeruginosa* PA14. Transductants were selected on M9-agar plates supplemented with 25 μM citrate and 1 mM MgSO_4_ and 75 μg/mL tetracycline (Tc). Individual colonies were repurified on LB-agar plates containing 75 μg/mL Tc. Putative *lasR* deletion mutants generated after the second recombination event were enriched by carbenicillin-treatment as described previously (Voisard et al., 2007). Surviving cells, which were Tc-susceptible were screened by PCR for loss of a 300 bp fragment in the *lasA* gene. One of three clones was selected for further analysis.

### Inhibition and Lytic Activity of *P. aeruginosa* Supernatants on *S. aureus*

We measured both inhibition of the *S. aureus* growth as well as lysis of *S. aureus* cells by *P. aeruginosa* supernatants.

The inhibition capacity of *P. aeruginosa* supernatants on *S. aureus* growth was tested in liquid. Overnight cultures of *P. aeruginosa* grown in MH-broth were centrifuged and the resulted supernatants were filtered (0.22 μm, Millipore). An overnight culture of *S. aureus* strain COL was adjusted to an OD_600_ of 0.1 in MH-broth. 100 μL of this bacterial suspension was incubated with 100 μL of *P. aeruginosa* supernatant. All mixtures were incubated in triplicate at 37°C with intermittent shaking and OD_600_ was recorded for 10 h in a plate reader (BioTek Synergy H1).

The lytic activity of *P. aeruginosa* overnight culture supernatants on *S. aureus* was tested on LB medium, supplemented or not with 1 mM uracil. The overnight cultures of *P. aeruginosa* were adjusted to the same OD_600_ before pelleting cells by centrifugation (6,000 × *g*, 10 min). Supernatants were filtered (0.22 μm) and used immediately. An overnight culture of *S. aureus* strain COL was adjusted to OD_600_ = 1 in 0.02 M Tris-HCl pH 7.5 and cells were inactivated by heating at 95°C for 10 min. 100 μL of the *S. aureus* bacterial suspension was incubated with 100 μL of filtered *P. aeruginosa* supernatants. Mixtures were incubated at 37°C with intermittent shaking and OD_600_ was recorded during 8 h in a plate reader (BioTek Synergy H1).

### Quantification of PQS Production

To test whether *S. aureus* affects PQS production by *P. aeruginosa*, an overnight culture of *S. aureus* strain COL was adjusted to an OD_600_ = 0.1 in LB medium. 190 uL of this dilution was deposited in a 96 well plate and incubated for 2 h without shaking. During this incubation, *P. aeruginosa* overnight cultures were centrifuged (6,000 rpm, 3 min) and the pellet adjusted to an OD_600_ = 2 in 0.9% NaCl. 5 μL of this cell suspensions of the *P. aeruginosa* PA14 and PA14 carB mutant were combined with 5 μL of the cell suspension of the PQS-indicator strain PA14 Δ*pqsA pqsA*::*lux* culture (Fletcher et al., 2007) and added to the 190 μL of the pre-incubated *S. aureus* cultures. The microtiter plate was incubated at 37°C with intermittent shaking and optical density at 600 nm and luminescence were recorded after 6 h. Experiments were done in duplicate.

### Growth Competition Assay with Supernatants of Competitor Cultures

*Pseudomonas aeruginosa* cultures were also spotted on MH-plates seeded with 500 μl of filtered supernatants from overnight competitor cultures. Overnight (18 h) cultures of competitor bacteria were centrifuged 10 min at 6,000 rpm. Supernatants were filtered (0.22 μm) and 500 μL of the filtrate was spread on agar plates. *P. aeruginosa* cultures were prepared as described above.

## Results

### Generation of the *P. aeruginosa* PA14 Transposon Insertion Library and Selection of Mutants Affected in their Capacity to Compete with other Respiratory Pathogens

**FIGURE 1:**
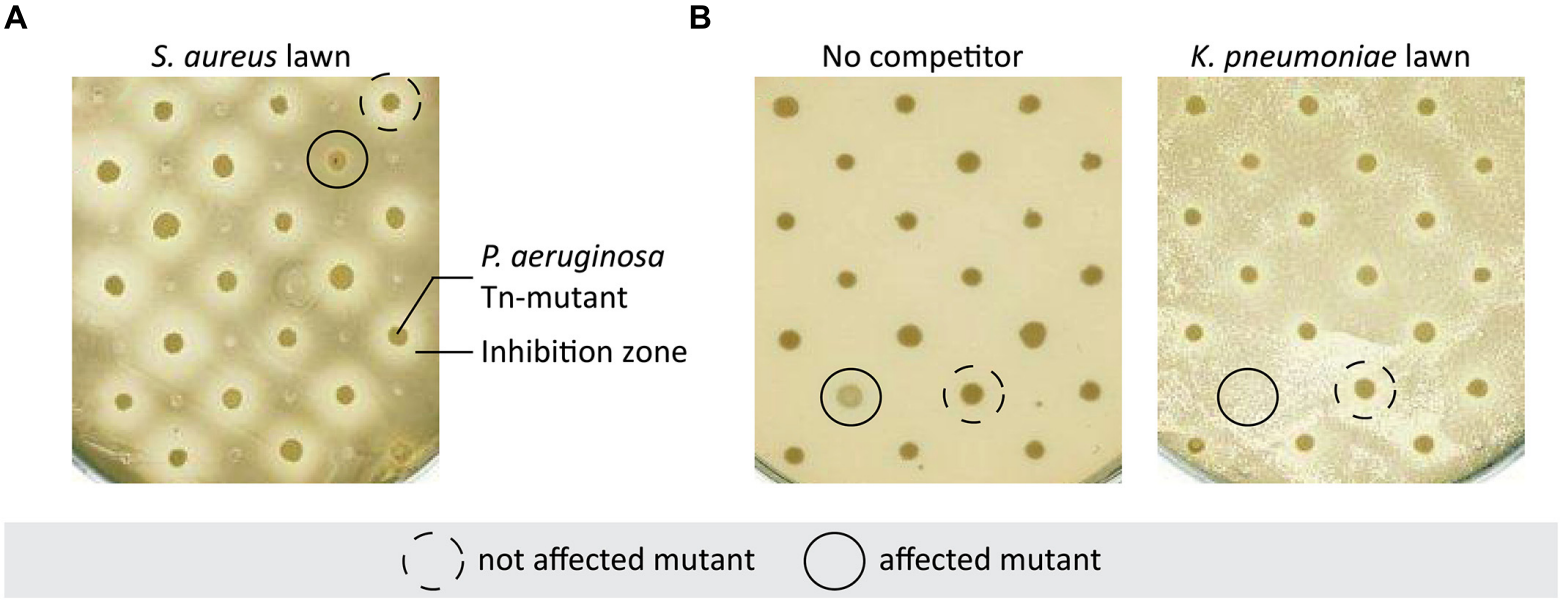
**Selection of *Pseudomonas aeruginosa* mutants affected in competition with bacterial pathogens. (A)** *P. aeruginosa* Tn-mutants were spotted on *Staphylococcus aureus* bacterial lawn on MH-agar plate. This panel shows an example of a mutant displaying an altered inhibition zone compared to the others, indicating that this mutant is not able to compete with *S. aureus*. **(B)*** P. aeruginosa* Tn-mutants were spotted on LB agar plate with or without *Klebsiella pneumoniae*. The unbroken line shows a mutant drastically affected by the presence of *K. pneumoniae* whereas it had a normal growth on plate without competitor.

We generated a library of 2,288 transposon insertion mutants in *P. aeruginosa* strain PA14. Three bacterial pathogens also commonly found in the lungs of CF patients were selected for the initial competition screening: *S. aureus*, *B. cepacia*, and *K. pneumoniae*. The PA14 mutant library was screened for: (i) an alteration of the inhibition zone produced by *P. aeruginosa* on a lawn of *S. aureus* (**Figure 1A**), and (ii) an alteration of *P. aeruginosa* growth in the presence of *B. cepacia* and *K. pneumoniae* (**Figure 1B**). Based on this screening, 64 mutants were retained displaying an altered inhibition zone (35 mutants) or growth (25 mutants) or both (4) in the presence of competitors when compared to wild-type PA14. Each of the selected mutants was re-tested in quadruplicates and four yielded reproducible phenotypes. Two mutants showed a reduction of the inhibition zone on a lawn of* S. aureus,* while the two others were affected in growth on lawns of *K. pneumoniae* or *B. cepacia*. Growth of these mutants was not affected on agar plates in the absence of competitors (data not shown).

### Role of the *P. aeruginosa pqs* Pathway During Competition with *S. aureus*

**FIGURE 2:**
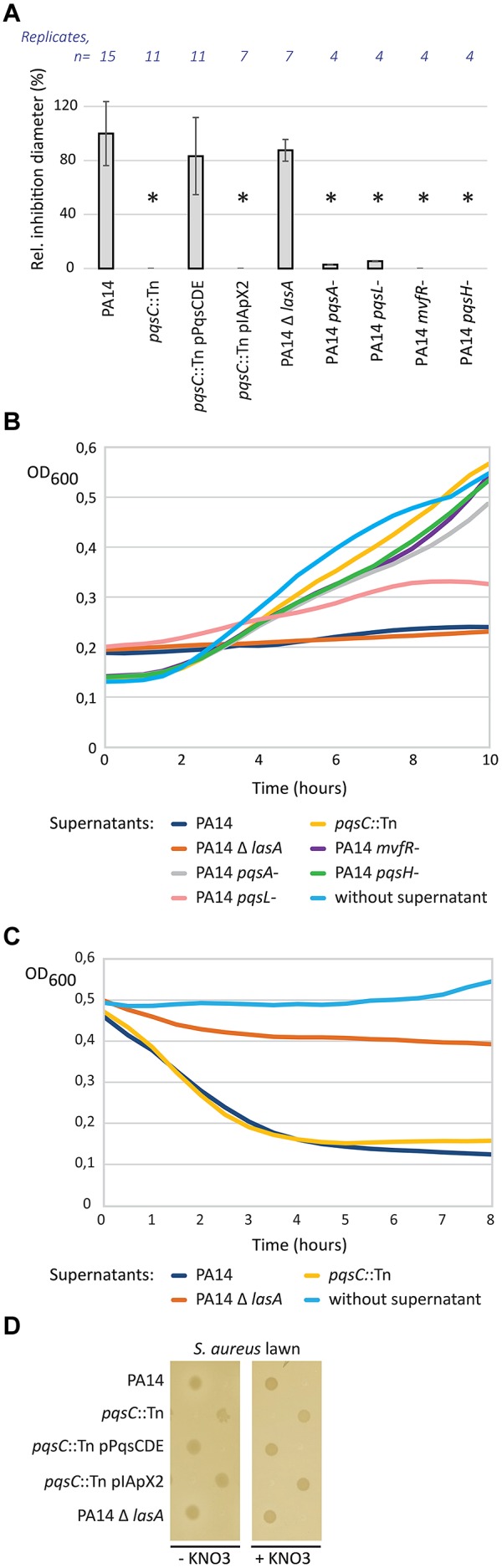
**Role of the *pqs* pathway of *P. aeruginosa* in direct competition with *S. aureus*. (A)** Effect of *pqsC* disruption on the inhibition of *S. aureus* growth. Spots of *P. aeruginosa* cultures were deposited on LB agar plates seeded with a lawn of *S. aureus* strain COL. Inhibition zone diameters were measured, normalized against the diameter of the corresponding spots and expressed as a percentage against the wild-type PA14 values. Error bars are standard deviations calculated on at least four replicates. Statistical significance was determined by using a *t*-test with unequal variances (^∗^*p* < 0.01). **(B)** Inhibition activity of *P. aeruginosa* supernatants on *S. aureus* growth. Supernatants of *P. aeruginosa* overnight cultures were incorporated on *S. aureus* cells. *S. aureus* growth (OD_600_) was monitored during 10 h at 37°C in presence to these supernatants. **(C)** Lytic activity of *P. aeruginosa* supernatants on inactivated *S. aureus* cells. Supernatants of *P. aeruginosa* overnight cultures were incorporated on heat-inactivated *S. aureus* cells. OD_600_ was monitored during 8 h at 37°C in presence to these supernatants to evaluate *S. aureus* lysis. **(D)** Role of the *pqs* pathway in hypoxic conditions. Spots of *P. aeruginosa* cultures were deposited on LB agar plates cover by a *S. aureus* lawn. Plates were incubated during 48 h in hypoxic conditions.

We determined the transposon insertion site in the *P. aeruginosa* clone most affected in inhibition of *S. aureus* growth, by a two-step semi-random PCR. The transposon was inserted at nucleotide position 136 of the *pqsC* gene, which belongs to the *pqsABCDE* operon (Xiao et al., 2006b). A *pqsC* mutant is deficient for the production of 4-hydroxy-2-alkylquinolines (HAQs; Dulcey et al., 2013). We complemented our *pqsC* transposon mutant by introducing a plasmid-encoded copy of the* pqsCDE* genes from PA14. The complemented mutant showed a partial restoration of the wild-type inhibition zone (**Figure 2A**). Because* pqsC* is part of a multigene operon, we tested the phenotype of strains mutated in other genes belonging to the PQS biosynthesis pathway. As expected, PA14 derivatives mutated in *pqsA* or* mvfR* (*pqsR*), both deficient in HAQ biosynthesis, did not inhibit *S. aureus* growth (**Figure 2A**). The same phenotype was observed for the *pqsH* mutant.

To test whether these phenotypes were limited to growth on solid medium, supernatants of *P. aeruginosa* overnight cultures were incubated together with a cell suspension of the *S. aureus* strain COL. Whereas supernatant of wild-type PA14 inhibited growth of *S. aureus*, supernatants from *pqsA*, *pqsC*, *mvfR,* and *pqsH* mutants showed no inhibitory activity. In agreement with the data obtained on solid medium, the *pqsL* mutant showed a partial inhibition of *S. aureus* growth in liquid medium (**Figure 2B**).

The LasA staphylolytic protease of *P. aeruginosa* cleaves the pentaglycine cross-links in the peptidoglycan of *S. aureus* leading to cell lysis (Kessler et al., 1993). We therefore tested the supernatant of a *lasA* mutant in competition with *S. aureus*, which did not alter the wild-type inhibition profile comparable to the one of the wild-type PA14 in our model (**Figure 2B**). These results demonstrate that the observed inhibition profile is not the consequence of the expression of LasA, but can be associated to the *pqs* pathway activity in wild-type PA14. To further distinguish between inhibition and lytic activity, we performed the same experiment on heat-inactivated staphylococcal cells (**Figure 2C**). Optical density related to *S. aureus* cells rapidly decreased after addition of PA14 supernatants. We observed the same profile with the *pqsC* mutant, indicating that HAQs do not contribute to *S. aureus* cell lysis but to growth inhibition. In contrast, PA14 Δ *lasA* was affected in its capacity to lyse *S. aureus* as expected from previous reports.

Because products of the PQS pathway are known to suppress growth in Gram-positive bacteria by inhibiting the respiratory chain in aerobic condition, we hypothesized that this phenotype may be abolished under hypoxic conditions. As expected, we observed no inhibition zone even for PA14 under hypoxic conditions (**Figure 2D**). Because nitrate is a key compound for the growth of *P. aeruginosa* under hypoxic conditions, we performed the experiment without and with addition of KNO_3_ as terminal electron acceptor. However, addition of KNO_3_ had no effect on the phenotype. This suggests that HAQs are unlikely to play a role in *S. aureus* growth inhibition under hypoxic conditions.

Taken together our experiments suggest that the *lasA* gene product is responsible for the lytic activity of *P. aeruginosa*, while HAQs are responsible for growth inhibition of *S. aureus* under aerobic conditions.

### Impact of the *carB* Mutation During Direct Competition between *P. aeruginosa* and *S. aureus*

**FIGURE 3:**
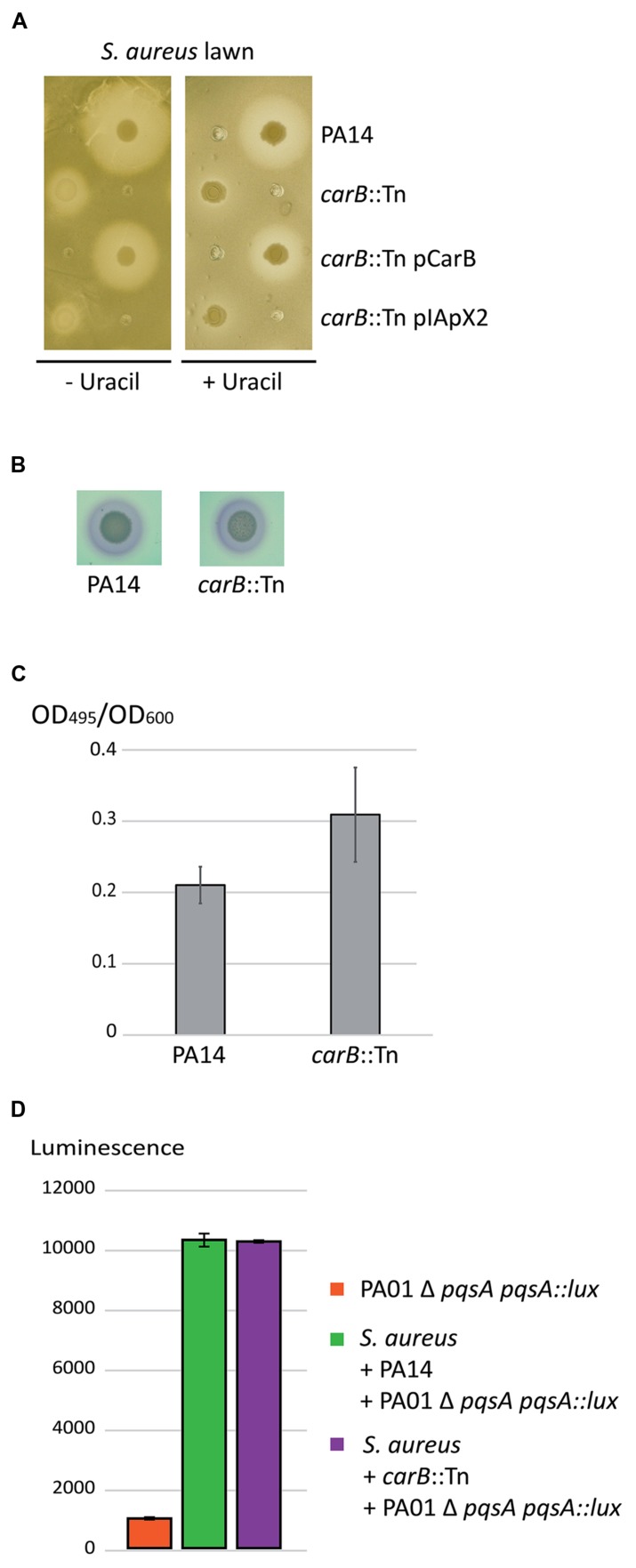
**Role of the *P. aeruginosa carB* gene during competition with *S. aureus*. (A)** Spots of *P. aeruginosa* cultures were deposited on LB agar plates seeded with a lawn of *S. aureus* strain COL. **(B)** Rhamnolipid production by the wild-type PA14 and the *carB*-disrupted PA14 strains. No difference was observed between the two strains within duplicates indicating that the Rhl *quorum-sensing* (QS) is not affected in the mutant. **(C)** Elastase production by the wild-type PA14 and the *carB*-disrupted PA14. Error bars indicate standard deviations calculated from three experimental replicates. **(D)** Quantification of PQS production during competition with *S. aureus*. The *carB* mutant and the wild-type PA14 strains were both incubated with the competitor and with the PAO1 Δ *pqsA pqsA*::*lux* reporter strain. PQS production was evaluated by quantification of the luminescence produced by the reporter strain in response to the amount of PQS in the medium. Luminescence was quantified after 6 h of incubation at 37°C. Error bars are standard deviations calculated on duplicate.

The second mutant affected in its capacity to inhibit growth, and therefore to compete with *S. aureus,* harbored the transposon inserted at position 1,632 of the *carB* gene (PA14_62910), encoding the large subunit of the carbamoyl phosphate synthase (Winsor et al., 2011). CarB is involved in the pyrimidine pathway leading to uracil synthesis. Indeed, supplementation with uracil restored growth of the *carB* mutant, but not the *S. aureus* growth inhibition (**Figure 3A**). Recently, *pyrF*, another gene required for uracil and pyrimidine synthesis was shown to affect the *quorum-sensing* (QS)-circuit in strain PA14 (Ueda et al., 2009). We therefore tested the QS-dependent virulence factor production in the *carB* mutant. However, we observed no difference in rhamnolipid (**Figure 3B**) and elastase production (**Figure 3C**) between the *carB* mutant and the wild-type PA14. To determine if the effect observed with the *carB* mutant during competition with *S. aureus* was due to an alteration of the *pqs* pathway we measured the production of PQS by quantifying luminescence produced by the PAO1Δ*pqsA pqsA*::*lux* reporter strain. The quantity of luminescence produced was identical between the *carB* mutant and the wild-type PA14 strain, indicating that *carB* does not affect the PQS production (**Figure 3D**) during competition with *S. aureus*. These results suggest that *carB* is required for inhibition of *S. aureus* growth, through a QS-independent mechanism.

### Role of the *carB* Gene During Competition between *P. aeruginosa*, *B. cepacia,* and *K. pneumoniae*

**FIGURE 4:**
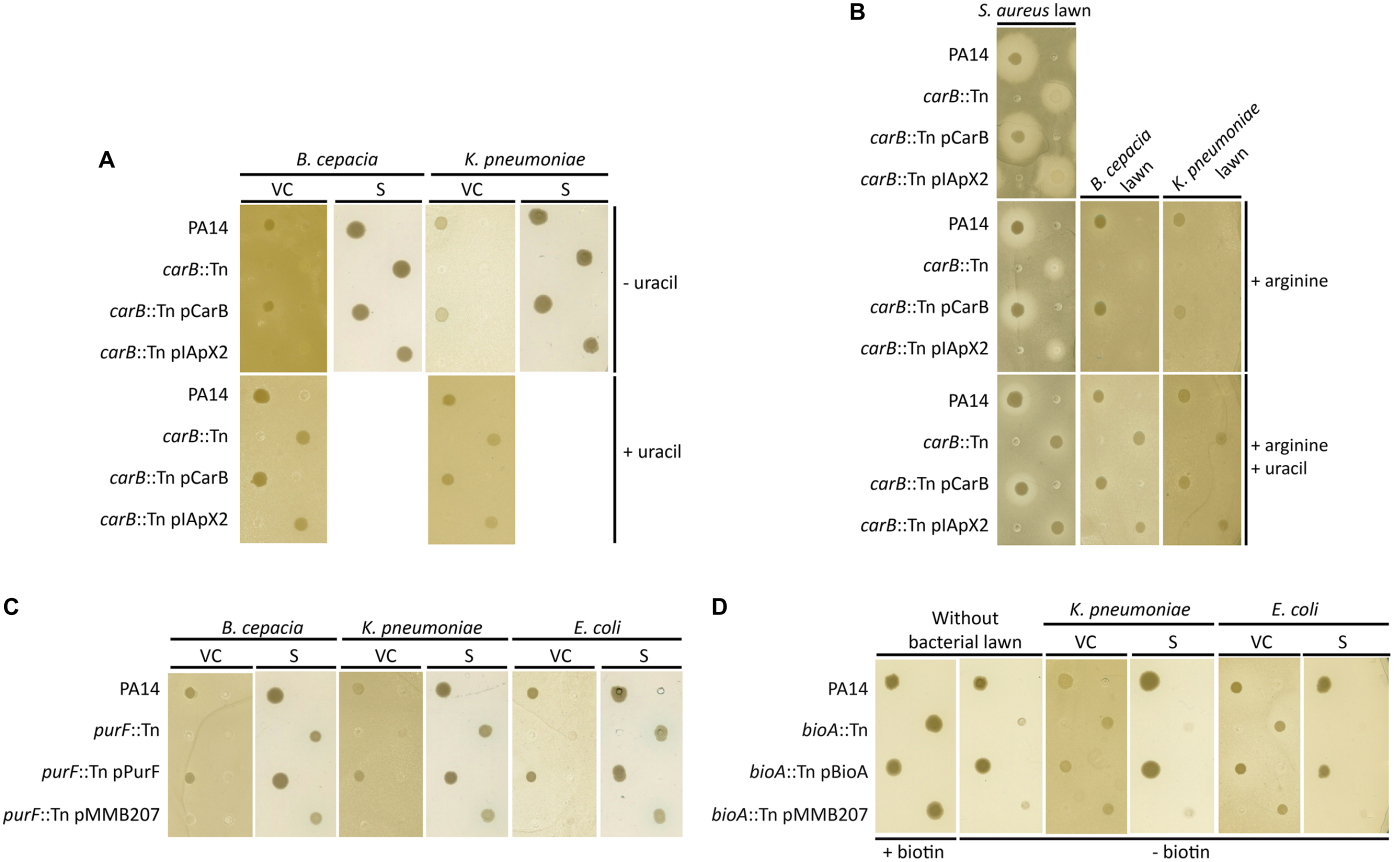
**Role of the *carB*, *purF* and *bioA* mutations during bacterial interactions between *P. aeruginosa* and other respiratory pathogens. (A–D)** Spots of *P. aeruginosa* cultures were deposited on LB agar **(A,B)** or MH agar plates **(C,D)** seeded with a lawn of *S. aureus*, *K. pneumoniae*, *B. cenocepacia*, or *E. coli*. Bacterial lawns were composed by viable cells (VC) from overnight cultures or by supernatants (S) of filtrated overnight cultures.

**FIGURE 5:**
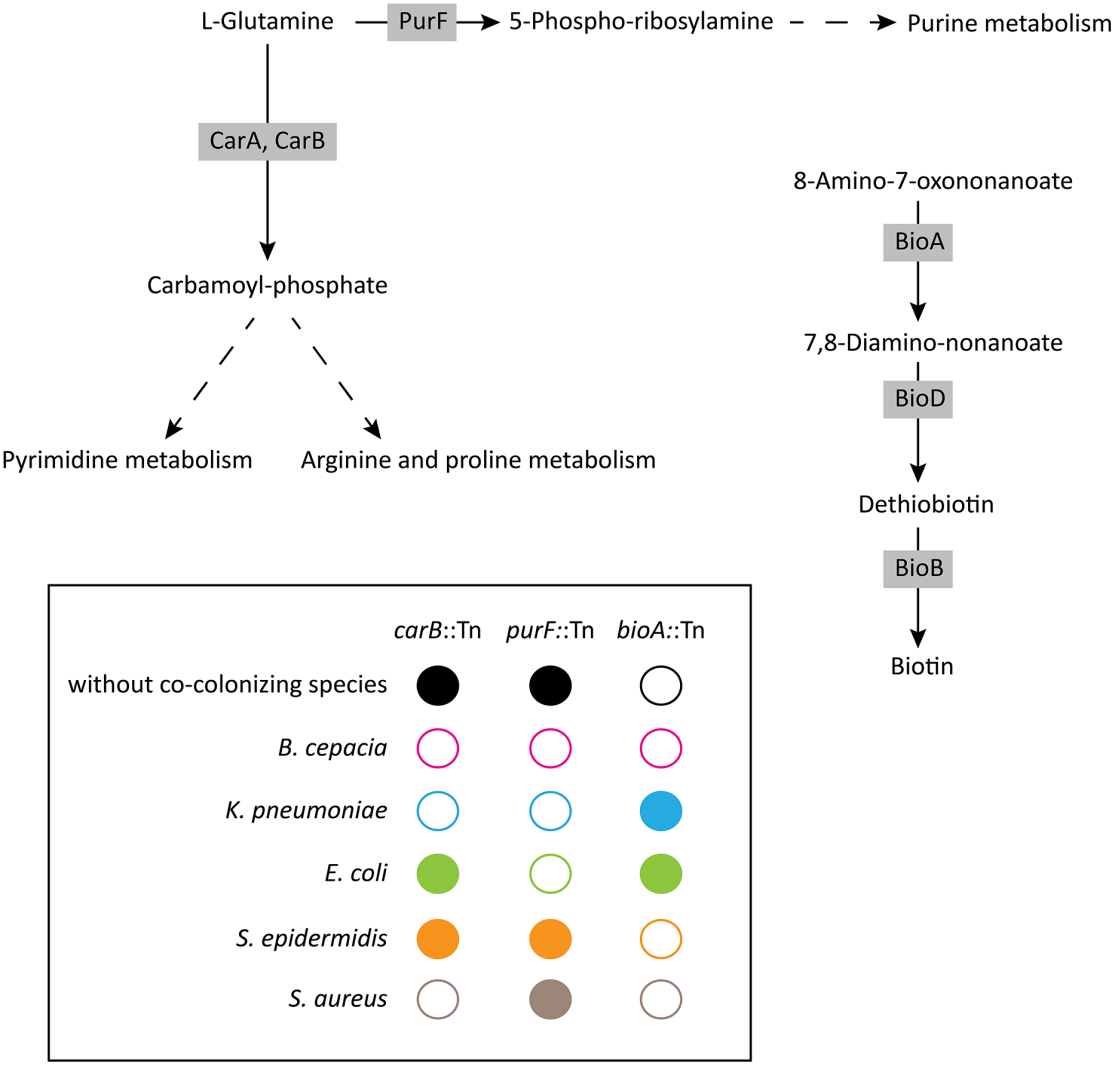
**Schematic summary of the growth phenotypes displayed by the *carB*, *purF,* and *bioA* mutants during interactions with other respiratory pathogens.** Metabolic pathways are based on the Kyoto Encyclopedia of Genes and Genomes databases. Open circles represent no growth, whereas filled circles indicate growth of *P. aeruginosa* mutants.

Two transposon mutants of *P. aeruginosa* were affected in their growth in the presence of *B. cepacia* and* K. pneumoniae* as competitors. One of these mutants was the *carB* mutant described above. This growth defect was complemented by the addition of a plasmid-encoded copy of *carB*, as well as by the addition of uracil (**Figure 4A**). Remarkably, growth of the *carB* mutant was not affected when competing with *E. coli* and *S. epidermidis* (data not shown). In contrast the addition of arginine alone, the second final product of this metabolic pathway (**Figure 5**), had no impact on the growth of the *carB* mutant (**Figure 4B**). To determine whether soluble factors secreted by the competitors could be involved, we plated supernatants of bacterial competitors on LB-agar plates prior to *P. aeruginosa* spotting. Culture supernatants of *B. cepacia* and *K. pneumoniae* did not affect the growth of the *carB* mutant, suggesting that the presence of metabolically active competing bacteria is required to repress growth of this mutant (**Figure 4A**). Taken together, these data demonstrate that the *carB* gene, and by analogy the uracil/pyrimidine biosynthesis, is an essential metabolic pathway for the competition of *P. aeruginosa* against *B. cepacia*, *K. pneumoniae,* and *S. aureus*.

### Effect of the *purF* Mutation on the *P. aeruginosa* Growth in Presence of *B. cepacia*, *K. pneumoniae,* and *E. coli*

The second mutant with a growth defect in the presence of competitors had the transposon inserted at position 264 of the *purF* (PA14_23920) gene, encoding an amidophosphoribosyl transferase. This protein is a key enzyme of purine metabolism, supplying nucleotides for DNA and RNA synthesis (Sampei and Mizobuchi, 1988). The *purF*::Tn mutant did not grow when cells were spotted on plates containing lawns of *B. cepacia*, *K. pneumoniae,* and *E. coli* (**Figure 4C**). This alteration was complemented by the addition of a plasmid-encoded copy of the *purF* gene. In contrast, the *purF* mutant showed normal growth in the presence of *S. epidermidis* and *S. aureus* (data not shown). As observed above for the *carB* mutant, culture supernatants of competing bacteria did not affect growth of the *purF*-disrupted strain (**Figure 4C**). This suggests that the *purF* gene is essential for *P. aeruginosa* growth in presence of the *B. cepacia*, *K. pneumoniae,* and *E. coli.*

### Role of the Biotin Synthesis Pathway on Beneficial Interactions between *P. aeruginosa* and other Respiratory Pathogens

To determine if growth of *P. aeruginosa* can be promoted by the presence of other bacterial pathogens, we screened the transposon library for mutants that grew better in the presence of a competitor than in its absence on rich medium. We identified one transposon mutant that grew poorly on MH-agar, but showed wild-type growth in the presence of *K. pneumoniae* and *E. coli* (**Figure 4D**). This growth “enhancement” was not seen in the presence of *B. cepacia*, *S. epidermidis,* and *S. aureus* bacterial lawns (data not shown). We identified the transposon insertion in this mutant at position 474 of the *bioA* (PA14_05460) gene, encoding an adenosylmethionine-8-amino-7-oxononanoate aminotransferase, involved in the biotin biosynthesis pathway (Stoner and Eisenberg, 1975). Indeed the addition of biotin, as well as extra-chromosomic complementation with the *bioA* gene, restored growth of the *bioA*-disrupted mutant (**Figure 4D**). Finally, growth of the *bioA* transposon mutant was not restored by supernatants of *K. pneumoniae* and *E. coli*, suggesting that the presence of metabolically active cells of these organisms is required to complement the growth deficit (**Figure 4D**).

## Discussion

Our study identified metabolic pathways involved in either detrimental or beneficial interactions between *P. aeruginosa* and four bacterial respiratory pathogens. By screening a transposon library of 2,288 mutants in PA14, we identified mutants displaying altered growth inhibition, or growth phenotypes in the presence of co-colonizing bacterial species. We found that both *pqsC* and *carB*, responsible, respectively, for HAQ and uracil/pyrimidine synthesis, play an essential role in the direct competition of *P. aeruginosa* with *S. aureus*. Secondly, we identified *carB* and *purF*, the latter involved in purine synthesis, as essential for exploitative competition against *K. pneumoniae* and *B. cenocepacia* (**Figure 5**). Finally, we found that co-colonizing strains may also have beneficial effects, as illustrated by growth restoration of the *P. aeruginosa bioA* mutant by *K. pneumoniae* and *E. coli* (**Figure 5**).

Studies on microbial interactions have already revealed the role of the *Pseudomonas* quinolone signal (PQS) biosynthesis pathway in the inhibition of *S. aureus* growth. It has been demonstrated that 2-heptyl-4-quinolone *N*-oxide (HQNO), one of the final products of the *pqs* pathway, suppresses growth of Gram-positive bacteria, but not of Gram-negative bacteria (Machan et al., 1992). In aerobic conditions, HQNO binds to quinone-reacting cytochromes and inhibits respiratory electron transfer from quinone to cytochromes (Magalon et al., 1998; Toyofuku et al., 2008). The present study confirms the role of the PQS-biosynthesis pathway in the direct competition with *S. aureus*. Indeed, *pqsC* acts at the beginning of the metabolic PQS-pathway and its inactivation leads to the absence of all HAQs including PQS, 2-heptyl-4-quinolone (HHQ), as well as their N-oxide derivatives. The *pqsC* mutant identified in our screening was unable to inhibit growth of *S. aureus* in rich medium. The wild-type phenotype was partially restored in the *pqsC* mutant by complementation with plasmid-encoded copies of the *pqsCDE* genes. The partial complementation can be explained by a gene dosage effect due to overexpression from the constitutive promoter present on the vector plasmid pIApX2. In addition, we demonstrated that the PQS-pathway does not play a role under hypoxic conditions. This result was not unexpected since under hypoxic conditions, the aerobic respiratory chain is not functional and cannot be inhibited by HQNO (Machan et al., 1992). Furthermore, HQNO and PQS seem to be produced only under aerobic conditions due to the oxygen requirement of the PqsH mono-oxygenase to catalyze the final conversion of HHQ to PQS (Schertzer et al., 2010). This suggests that in the thickened mucous layers in the airways of CF patients, where low oxygen concentrations are prevailing (Worlitzsch et al., 2002), HQNO may play only a limited role in interference between *P. aeruginosa* and *S. aureus*. We can also hypothesize that the role of the PQS-system on *S. aureus* is limited in mixed biofilms, representing microaerophilic growth conditions. Nevertheless, HHQ and PQS were identified in sputum samples from CF patients (Collier et al., 2002; Palmer et al., 2005) indicating that maybe, in the more oxygenated upper respiratory tract, HQNO might play a more prominent role in growth inhibition of Gram-positive bacteria.

Our results further suggest that the N-oxide derivatives of HAQs are responsible primarily for the *S. aureus* growth inhibition. As described above, the *pqsH* mutant was affected in the conversion of HHQ to PQS. PQS contributes to the positive feedback regulation of the pqsA-D genes via PqsR. Thus, a defect in PQS production should lead to the absence (or major decrease) of HHQ and HQNO in a *pqsH* mutant. In addition our results showed a small residual activity of the *pqsL* mutant, which displayed only a weak inhibition of *S. aureus* growth both on agar and in liquid. PqsL is a putative mono-oxygenase converting HHQ to HQNO, and PQS to its N-oxide derivative (Lepine et al., 2004). A *pqsL* mutant was shown to produce increased amounts of PQS (D’Argenio et al., 2002), and maybe also of other HAQs, suggesting that the non-*N*-oxide derivatives may also display a weak growth inhibitory effect on *S. aureus*.

In addition to the role of PQS, we demonstrated that the *P. aeruginosa carB* gene is required for direct competition with *S. aureus*. Without uracil, the *carB*-disrupted strain exhibited an alteration in its capacity to inhibit *S. aureus* growth. However, the growth of this mutant appeared to be slightly affected. Nevertheless, when we added uracil in the medium, the growth of the *carB*-disrupted strain was totally restored, but the inhibition of *S. aureus* growth was still diminished. Previous reports suggested that uracil influences all three QS*-*systems in *P. aeruginosa* (i.e., *las*, *rhl*, *pqs*; Ueda et al., 2009). Since uracil is the final product of the biosynthesis pathway containing the *carB* gene, we tested if the phenotype exhibited by the *carB*-disrupted strain can be linked to QS. However, phenotypic analysis of QS-dependent traits showed that none of the three QS-systems was affected in the *carB*-disrupted strain. This indicates that a QS-unrelated mechanism was responsible for reduced inhibition of *S. aureus* growth in the *carB*-disrupted strain.

Interestingly, the *carB* gene also appeared essential for the exploitative competition between *P. aeruginosa* and *S. aureus*, as well as with *B. cepacia* and *K. pneumoniae*. These results suggest that *carB* is involved in the competition for resources between *P. aeruginosa* and these three competitors. CarB is an enzyme catalyzing the transformation of L-glutamine into carbamoyl-phosphate, an intermediate in the pyrimidine, arginine, and proline metabolisms (**Figure 5**). We show that the phenotype of the *carB*-disrupted strain can be restored by the addition of uracil, but not of arginine, demonstrating that the role of the *carB* gene in the exploitative competition can be associated to the pyrimidine biosynthesis pathway.

In addition to *carB*, the *purF* gene also appeared to be an essential gene for exploitative competition. Interestingly, growth of the *purF* mutant was not affected in the presence of Gram-positive bacteria (*S. aureus* and *S. epidermidis*) but was severely affected in grow in the presence of the Gram-negative strains tested (*B. cenocepacia, K. pneumoniae, E. coli*). The *purF* gene encodes an amidophosphoribosyltransferase involved in the conversion of L-Glutamine into 5-Phospho-ribosylamine, a primary product of the purine metabolism (**Figure 5**). Taken together, these results highlight the essential role of nucleic acid biosynthesis pathways for exploitative competition. Interestingly, the purine and pyrimidine synthesis pathways were also shown to be important for colonization of the mouse intestine by *E. coli* (Vogel-Scheel et al., 2010), an environment where invading organisms have to compete with resident microbial flora.

Finally, we identified beneficial interactions between *P. aeruginosa* and other lung co-colonizing species via the biotin synthesis pathway. Whereas a *P. aeruginosa bioA*-disrupted mutant was unable to grow on rich medium, the presence of *K. pneumoniae* and *E. coli* complemented the growth defect of this strain (**Figure 5**). In other words, the presence of *K. pneumoniae* and *E. coli* can be beneficial to *P. aeruginosa* when biotin supply is limited. However, the molecular basis of this beneficial interaction remains to be elucidated.

## Conclusion

Understanding the interactions between the various bacterial communities colonizing the CF-airways is of considerable importance as interactions can potentially affect the metabolism of pathogens, alter the population structure and eventually influence disease progression. It is clear from our experiments that complex interactions take place between CF-lung colonizing species which may lead to profound changes in bacterial community structures in CF-patients. Detrimental and beneficial interactions not only modulate the richness and the diversity of species but also create a complex environment in which bacterial species need to adapt to co-colonizing species aiming to increase their relative fitness. The metabolic pathways involved in these complex interactions could potentially be exploited to manipulate microbial population structure to improve the clinical outcome of chronic infectious.

## Conflict of Interest Statement

The authors declare that the research was conducted in the absence of any commercial or financial relationships that could be construed as a potential conflict of interest.
